# State of the Art Modelling of the Breast Cancer Metastatic Microenvironment: Where Are We?

**DOI:** 10.1007/s10911-024-09567-z

**Published:** 2024-07-16

**Authors:** Mia Nuckhir, David Withey, Sara Cabral, Hannah Harrison, Robert B. Clarke

**Affiliations:** Breast Biology Group, Manchester Breast Centre, Division of Cancer Sciences, Oglesby Cancer Research Building, Faculty of Biology, Medicine and Health, University of Manchester, Manchester, M20 4GJ UK

**Keywords:** Breast cancer metastasis, Bone, Lung, Liver, Brain, Lobular breast cancer

## Abstract

Metastatic spread of tumour cells to tissues and organs around the body is the most frequent cause of death from breast cancer. This has been modelled mainly using mouse models such as syngeneic mammary cancer or human in mouse xenograft models. These have limitations for modelling human disease progression and cannot easily be used for investigation of drug resistance and novel therapy screening. To complement these approaches, advances are being made in ex vivo and 3D in vitro models, which are becoming progressively better at reliably replicating the tumour microenvironment and will in the future facilitate drug development and screening. These approaches include microfluidics, organ-on-a-chip and use of advanced biomaterials. The relevant tissues to be modelled include those that are frequent and clinically important sites of metastasis such as bone, lung, brain, liver for invasive ductal carcinomas and a distinct set of common metastatic sites for lobular breast cancer. These sites all have challenges to model due to their unique cellular compositions, structure and complexity. The models, particularly in vivo, provide key information on the intricate interactions between cancer cells and the native tissue, and will guide us in producing specific therapies that are helpful in different context of metastasis.

## Introduction

Most breast cancer originates in the epithelial cells of the adult mammary gland, where mutations in the DNA of oncogenes and tumour suppressor genes accumulate during proliferative cycles driven by ovarian hormones and pregnancy. Thus, major risk factors include reproductive status such as early menarche, late menopause, parous status, age at first pregnancy and months of breast feeding. Other major risk factors include mammographic breast density and a family history including heritable BRCA1/2 and other single gene mutations or polygenic risk factors [1, 2]. There are premalignant stages such as ductal or lobular carcinoma in situ (DCIS/LCIS) but metastasis is associated with invasive breast cancer of ductal or lobular histological sub-types. Both sub-types are thought to arise in the terminal ductal lobular units of the breast from the luminal epithelial cells that contains progenitors, hormone-sensing cells and cells that produce milk at lactation. Invasive ductal and lobular carcinomas (IDC/ILC) have different genetic mutations, for example, TP53 and CDH1 loss, respectively. IDC accounts for more than 80% of breast cancers and ILC for ~ 15% with some rarer histological subtypes for the remainder [3]. Four general classes of IDC can be distinguished by immunohistochemical (IHC) staining for hormone receptors for estrogen and progesterone (ER and PR) and the human EGF-related receptor-2 (HER2). These are ER + PR + /HER2-, ER + PR + or-/HER2 + , ER-PR-/HER2 + and ER-PR-/HER2- that distinguish the 4 molecular subtypes luminal A, luminal B, HER2 + and triple negative breast cancer (TNBC) defined by their pattern of expression of the PAM50 geneset [4]. The molecular sub-type defined by IHC of the primary tumour is used clinically to determine the therapy alongside information on tumour size, grade, and its spread to local lymph nodes (LN) or to other sites distal to the primary tumour.

Ninety-five percent of breast tumours are detected before spread beyond the breast and LN is apparent but metastasis to other tissues is the cause of recurrences in up to a third of breast tumours suggesting that cells have migrated from the primary tumour at an early stage before the primary tumour is surgically removed. Metastatic spread of tumour cells to tissues and organs around the body is the most frequent cause of death from breast cancer and is difficult to treat once it occurs. The most common sites of distant metastasis for IDC are the bone (~ 70%), followed by lung (~ 30%), liver (~ 25%) and brain (< 10%) and the molecular subtypes have differences in the metastatic patterns with luminal sub-types more likely to metastasise to bone, TNBC to liver and lung and HER2 + to the brain [5]. ILC have a metastatic pattern distinct from IDC and can be found in the ovaries, gastrointestinal tract and in the peritoneal ascites [6]. The seed and soil hypothesis of metastatic spread was proposed 150 years ago by Stephen Paget to explain that growth of metastatic tumours would be partly explained by the target tissue biology and partly by the biology of the cancer cells themselves [7]. More recently, we have learned more about the influence of the tumour cells on the metastatic tissue, both on its extracellular matrix (ECM) and cells, and the reciprocal signals that go between them. Thus, to discover more about the biology, treatment sensitivity and novel targets, we aim to model these interactions and the effects on the cell biology of both the metastatic tumour and the tissue cells. These models comprise in vitro, in vivo (mostly in mouse) and ex vivo approaches that encompass different tissue components of metastatic sites. Here we review bone, lung, liver and brain models and point to remaining gaps in knowledge and models that could be developed in the future to address these.

## Bone Metastasis

**Fig. 1:**
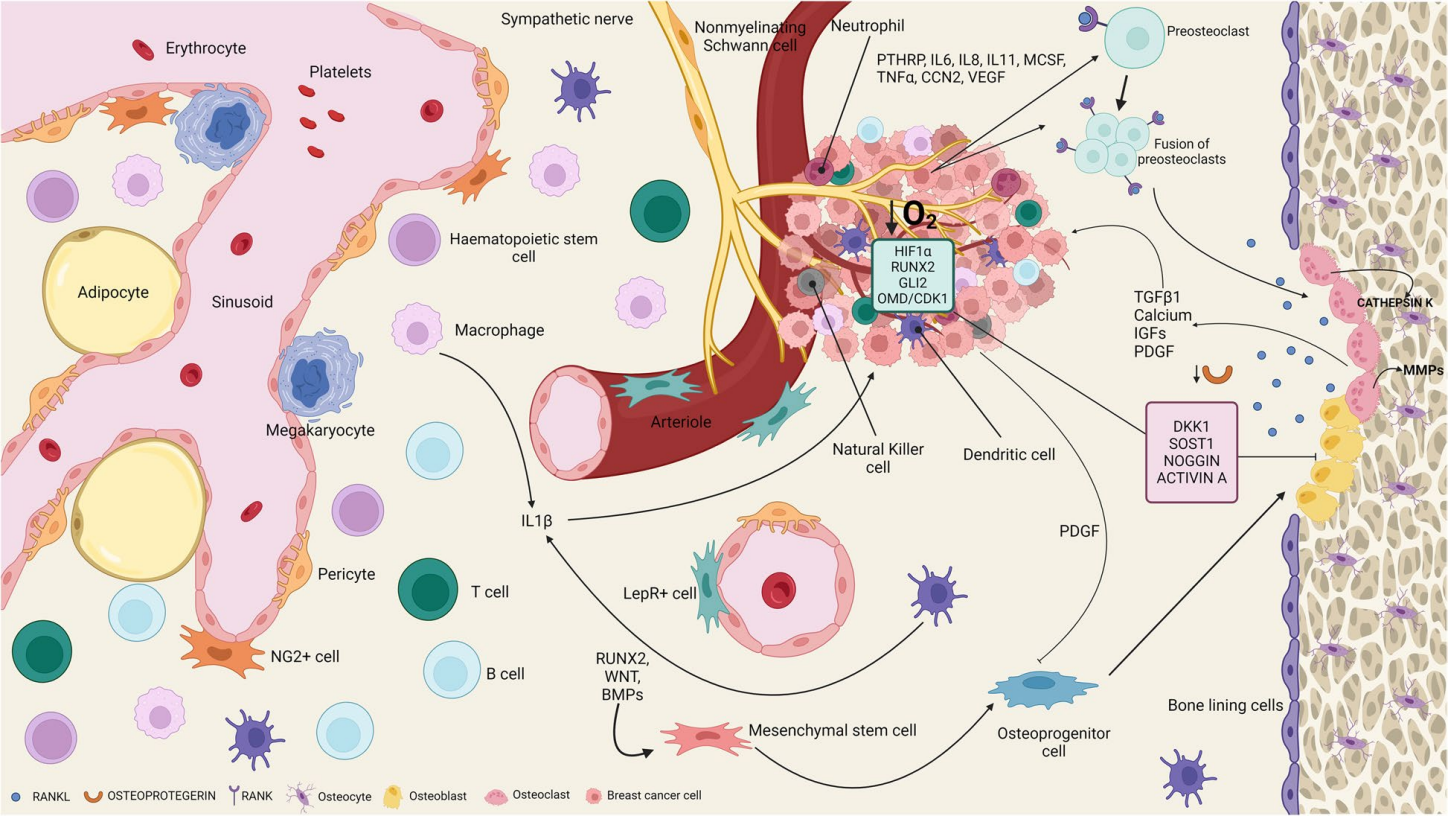
Breast Cancer Microenvironment in Bone. The Bone Marrow is a Complex Environment With Various Cell Populations. Vascular Endothelial Cells and Associated Stromal Cells Such As LepR + and NG2 + cells and Pericytes Are Important Regulators of Haematopoietic Stem Cell (HSC) Activity. Osteoblasts Line the Endosteal Surface of the Bone and Also Have Important Roles in HSC Maintenance. These Originate From the Differentiation of MSC. HSC Derive Various Cells Including Macrophages, Megakaryocytes, Dendritic Cells and B and T Cells. These Feedback and Help Regulate HSCs. Other Cells Are Present Such As Nonmyelinating Schwann Cells and Sympathetic Nerves. The Central Bone Marrow Has a Lower Oxygen Tension Than Other Tissues. The Central Bone Marrow is Quite Soft While the Endosteal Region is Stiffer. When Breast Cancer Cells Metastasise to Bone, They Take Advantage of the Various Cytokines Naturally Present in This Environment to Establish Themselves and Proliferate. Tumour Cells Also Undergo Various Changes to Become Better Adapted to the Bone Microenvironment. They Also Disrupt Bone Homeostasis By Stimulating Osteoclastogenesis and Inhibiting Differentiation to Osteoblasts As Well As Their Activity. This Induces the Formation of the Osteolytic Lesions Associated With This Disease. (DKK1 – Dickkopf 1; IGF – insulin-like growth factor; IL – interleukin; LepR + – Leptin receptor positive; M-CSF – macrophage-colony stimulating factor; MMPs – matrix metalloproteinases; NG2 +—neural-glial antigen 2 positive; OMD – Osteomodulin; PDGF – platelet-derived growth factor; PTHRP – parathyroid hormone-related protein; SOST1 – sclerostin; TGFβ1 – Transforming growth factor beta 1; TNF – tumour necrosis factor; VEGF – vascular endothelial growth factor)

Bone is a very dynamic tissue that is continuously undergoing remodelling which allows for normal skeletal structure and function. Homeostasis is coordinated by the balanced activities of osteoclasts and osteoblasts present on the surface of trabecular bone. Osteoclasts resorb and remove mineralised bone which is followed by the formation and deposition of new bone by osteoblasts. During the process of colonisation, breast cancer cells (BCCs) produce factors that overstimulate osteoclastogenesis, causing the formation of osteolytic lesions within the bone. Increased levels of bone resorption result in the release of growth factors – e.g., parathyroid hormone-related protein – from within the bone matrix that stimulate the growth of the BCCs while also further promoting osteoclastic activity. This establishes what is known as the “vicious circle of destruction”. However, it is important to note that bone is not just made up these components alone. Besides a very complex and rich ECM and the presence of hydroxyapatite, the bone marrow is present within the medullary cavity [8]. Haematopoiesis, osteogenesis, osteoclastogenesis and immune responses are regulated within this highly specialised organ. As a result, various cell types reside including haematopoietic stem cells, mesenchymal stem cells (MSCs) and immune cells, all of which have a described role in regulating bone metastasis (see review by [9]) (Fig. 1). This makes bone a unique metastatic environment due to containing various developmental and self-renewal signals such as members of the Wnt and Notch families [10]. Various models have been developed over the past few years that attempt to elucidate the mechanisms behind breast cancer’s preference for bone and how the BCCs disrupt bone homeostasis along with the relationships with native bone cells. As previously described, bone has two different environments: trabecular bone, the mineralised and stiffer component; the bone marrow which is softer and densely populated with different cell types. Both components play a very significant role in homing, survival, and colonisation of cancer cells. Therefore, great care should be taken to ensure that the overall structure and morphology of this microenvironment is appropriately considered.

### In Vitro Models: On-a-chip and Organoid Approaches

Microfluidic devices, also known as “organ-on-a-chip”, have presented themselves as a better solution when referring to in vitro models. Their main advantage is the ability to control and manipulate both gases and fluids within a small network of channels and chambers. This makes them more representative of native tissues as it replicates vascularisation and simulates shear stress, and more cell populations can be included thanks to the use of various segregating chambers. Breast cancer within bone marrow has successfully been modelled using this approach, specifically the perivascular and endosteal niches. This has been done mainly by incorporating resident bone marrow cells like MSCs, haematopoietic stem cells and endothelial cells within fibrin hydrogels [11]. Haematopoietic stem cells were able to maintain their multipotency while the addition of BCCs demonstrated successful migration and infiltration within the bone marrow-like environment. Other models have addressed the involvement of the immune system, an area of study that remains so far elusive. The activity and behaviour of neutrophils and macrophages has successfully been replicated within this type of system while maintaining features of metastatic behaviour [12, 13]. Out of all the in vitro models, microfluidic devices are by the far the most comprehensive attempts at modelling this tumour microenvironment. There are several other models that focus on the internal dynamics of bone marrow [14, 15]. While these do not include BCCs, they are nonetheless worth a mention as they could be adapted to recapitulate breast cancer colonisation within bone.

Organoids have established themselves as an alternative to in vivo models due to the ability of replicating the complex structure and functions of organs in 3D culture. They can contain multiple cell types that have been derived from stem cells, tissue-derived cells, and cancer cells [16]. Various breast cancer organoids have been established from patient cells that recapitulate disease heterogeneity with good success rates including some derived from metastatic lesions [17, 18]. Some bone metastasis organoids have also been recently developed from bone lesions [19]. Following next-generation sequencing, various cell populations were identified including osteoclasts and endothelial cells and potential therapeutic targets explored. However, it is also worth exploring the option of developing bone marrow organoids that can later be populated by BCCs. A bone marrow organoid has been recently developed that includes all the key components of the human bone marrow and replicates its structure, activity and function [20]. It would be of interest to combine both to allow the study of the cellular interactions while also providing a platform for drug screening.

Additive manufacturing techniques have been employed to address and replicate the structural aspects of the bone in vitro. Stereolithography has been employed to produce a highly porous scaffold with an elastic modulus close to that of native bone. Han et al*.* [21]*,* were able to reproduce the trabecular structure by scanning sections of the femur’s epiphysis using micro-computerised tomography. This material was compatible with MSC growth and osteogenic differentiation while also being capable of hosting patient-derived xenografted breast cancer cells (BCC) and maintaining an in vivo drug response. A biomimetic bone matrix can also be fabricated using stereolithography and an ink containing hydroxyapatite [22]. Both BCCs and bone marrow-derived MSC were seeded into this scaffold and demonstrated a behaviour that resembles native tumour conditions. Another layer of complexity was subsequently added to this model by including osteoblasts which were separated by a transwell system and recapitulated osteoblast inhibition and IL-8 secretion [23]. A different type of 3D printing is the process of bioprinting which gives the user the ability to generate scaffold-free tissue models that contain different cell types. Using bioinks containing both cancer and stromal cell types, it is possible to incorporate them in a spatially defined way that allows the deposition of ECM and the organisation of endothelial cells into complex networks. This method has been successfully applied in the context of breast cancer bone metastasis (BCBoM) [24, 25]. Others have focused on including hydroxyapatite within the structure as it is the main inorganic component of bone and has been shown to influence the behaviour of BCCs by favouring a more aggressive phenotype [26]. Hydroxyapatite-based polymers manufactured via salt-leaching have been developed to mimic the constitution of trabecular bone [27, 28]. The system not only allowed the modelling of colonisation, progression, and osteolytic lesion formation, but was able to induce the secretion of IL-8, a pro-osteoclastic cytokine [29]. Using a similar approach, Kar et al. created a 3D nanoclay scaffold that was posteriorly seeded with MSCs and BCC lines [30, 31]. The model showed much promise to study the pathogenesis of bone metastasis as well as a screening tool for therapies. A more recent development involved the fabrication of gelatin microribbons in order to establish a tri-culture model [32]. Here, both breast and prostate cell lines were used to study invasion, aggressiveness, changes in bone remodelling and drug responses. Osteoblasts and osteoclasts were also incorporated, allowing them to recreate osteolytic lesions. González Diaz and colleagues also demonstrated the possibility of integrating 3D models with single cell sequencing techniques. Overall, these models show much promise by mimicking the structural and inorganic properties of bone. However, they fail to include other cell types present within the bone matrix i.e., osteocytes and the bone marrow. Therefore, they represent a very simplistic view of this metastatic site.

Other scaffold-based approaches involve the use of hydrogels. Hydrogels are a highly hydrated 3D polymeric network that attempts to mimic the structure of a tissue’s ECM, making them biocompatible. Type I collagen has been widely used as a basis for developing models. Co-culture models using BCCs and bone cells within this matrix have been able to mimic some of the events that take place during colonisation [33]. Another approach involved inducing mineralisation of a collagen hydrogel to understand the role of hydroxyapatite in breast cancer stem cell behaviour [34]. They provide conclusive evidence of how the mineral content of bone within the osteogenic niche can influence the phenotype of BCCs. In addition to collagen, other hydrogel-based synthetic matrices have also been developed. Polyethylene glycol (PEG) hydrogels is one avenue that has been pursued for recreating the bone marrow microenvironment by adding tissue-specific peptides [35]. MSCs were compatible with PEG hydrogels as they proliferated and differentiated as expected in their native environment. This platform shows promise to be developed into a more comprehensive model of the bone marrow. A different PEG-based hydrogel was developed for the study of ER + breast cancer dormancy that contained an integrin-binding sequence and an enzymatically degradable linker to allow remodelling by cells [36]. This material has been since applied for studying dormancy within a bone marrow niche in the form of direct and indirect co-culture systems [37]. A more recent hydrogel technology uses self-assembling peptides. There are two different types; the α-helix and β-sheet – which are differentiated by the configuration they gain after spontaneous assembly. They have been used to culture different cell types and even for passaging patient-derived xenografts [38]. More importantly, they have been shown to be compatible with mimicking breast cancer behaviour at the primary site along with the growth of MSCs and osteoblasts [39–41]. Therefore, this demonstrates great promise as a material. Overall, hydrogels alone are capable of mimicking the bone marrow by including key ECM components and replicating its structure. However, supplementing it with a trabecular bone-like scaffold could elevate a model to be truly representative of bone.

### Ex Vivo Cultures

Bone tissue explants can be considered a more physiologically appropriate approach for mimicking BCBoM conditions. All the relevant cell types and ECM proteins are already present which allows a more comprehensive study of the behaviour of BCCs within the bone. For this method, human and mouse bones are typically employed. Human bones are typically harvested from patients undergoing hip replacement procedures which due to the high number of operations taking place every year presents itself as a good source. This approach has been described for the study of bone metastasis in breast cancer and has aided in studying the role of IL-1β and leptin [42–46]. Human subchondral bone discs with BCC lines can remain viable for a maximum of 4 weeks which permits long-term studies. Conversely, mouse calvarial bone can be used but they carry similar limitations as those previously described for the use of mouse models along with concerns surrounding ethics [47]. Furthermore, the choice of mouse bone can be considered clinically irrelevant as BCBoM does not typically occur to the skull.

### In Vivo

In vivo models of BCBoM have been developed to study the early stages of micrometastases, latency, colonisation, and osteolytic bone disease. Some genetically engineered mouse models of breast cancer have been employed, but most metastasize to the lungs and less to the bones [48]. The orthotopic transplantation of tumours has been used but very few develop bone metastases, except for bone-tropic 4T1 clones and the MSP-overexpressing PyMT transgenic mouse model [49, 50]. Other approaches include intra-tibial, intra-cardiac or intra-iliac artery injections [51–53]. Intra-tibial drilling has been shown to successfully deliver breast cancer cells to the bone but incurs damage and significant inflammation to the local tissue. Intra-cardiac injection was for some time the most widely used approach for inducing bone colonization but produces variable results. Thus, the intra-iliac artery approach was developed to selectively deliver cancer cells to the bone microenvironment very successfully [54]. However, these models present various disadvantages where human-derived tumours are employed, for example the lack of human ECM components, human secreted factors and an immune system. These lacking features prevent the study of the tumour interaction with the relevant microenvironment. In response to this, several humanisation approaches have been developed to overcome this from engrafting human haematopoietic cells to the implantation of human bone discs within mice [55–57]. These have allowed the formation of a more clinically relevant model, but the impact of the surrounding murine tissue upon the grafts remains to be elucidated.

## Lung Metastasis

The lungs are a pair of complex respiratory organs responsible for gas exchange. They are composed of approximately 40 different resident cell types, each with specific functions that aid the respiratory and non-respiratory roles of the tissue in the proximal and distal airways [58]. Breast cancer lung metastases (BCLuM) most commonly occur in women with the basal-like TNBC subtype. The median survival rate for patients with BCLuM is just 21 months and patients often suffer from a series of debilitating co-morbidities such as hemoptysis, lymphangitis, and pleural effusion [59]. Current models to study the metastatic lung microenvironment are lacking and limited by their ability to fully recapitulate the complex cellular composition and architecture of the tissue. Here, we review advanced models used to study BCLuM.

### In Vitro Models: On-a-chip and Organoid Approaches

On-a-chip microfluidic systems are miniaturised in vitro models that combine microfluidics with cell biology. Lung-on-a-chip microfluidic devices have been engineered to study the lung both as a primary tissue for pulmonary research, as well as a secondary tissue for metastasis research [60, 61]. On-a-chip devices have been used extensively in breast cancer research to interrogate different parts of the metastatic cascade. For example, invasion and extravasation in BCLuM have been modelled using normal human lung fibroblasts [61, 62]. The effects of sheer stress on circulating tumour cells as well as organotropism have also been modelled using on-a-chip devices with endothelial cell and organoid components [63, 64].

The multi-site on-a-chip models used primarily to study organotropism could prove particularly important for investigating the interactions between cancer cells and the metastatic lung microenvironment. In these devices, organ models representing the primary and metastatic sites are connected under applied fluid flow. These organ models vary in their complexity. For example, Firatligil-Yildirir et al*.*, [61] have used normal lung fibroblasts that self-assemble into 3D structures in Matrigel and Aleman and Skardal [63] have used bioengineered, hydrogel-based 3D lung organoids from a human lung epithelial cell line. Furthermore, Xu et al., [65] have designed a microfluidic chip housing a multicellular ‘lung organ’. In this system designed to study lung cancer metastasis, human bronchial and lung cancer cells are cultured together in a microchannel, separated from stromal macrophages, fibroblasts, and endothelial cells by a PDMS membrane. To model metastasis, cell lines representative of the sites of lung cancer metastasis form 3D cultures in connected chambers and physical expiration can be modelled by cyclical mechanical stretching and air exposure to replicate physiological features of the lungs [65]. It will be important to draw on models like these used to study primary lung cancers, as well as other cancers that metastasise to the lungs to advance current microfluidic models used in breast cancer research.

The main difficulty in using these microfluidic systems to study metastasis lies in the ability to fully recapitulate the primary and metastatic tissue microenvironments. Different cells with different culture conditions are required to accurately recreate the distinct tissue microenvironments, which can be difficult to achieve in a single device. However, owing to the great deal of flexibility in the design of these devices, they represent an exciting, evolving area of in vitro research.

Organoids are self-assembled 3D models that mimic the complex structure of specific organs. They contain various cell types, representative of the target organ and are produced from either embryonic or induced pluripotent human and mouse stem cells or primary human or mouse cells. Examples of lung organoids include but are not limited to epithelial broncho- and trachospheres and alveolar organoids containing AT1 or AT2 cells.

Few methods exist to generate lung organoids that represent the cellular complexity of both the distal and proximal lung microenvironments. In one example, Dye et al., [66] develop anterior foregut spheroids from human embryonic stem cells and directed these spheroids to a lung lineage via the expression of NKX2.1, a gene essential for lung organogenesis. Two months after transfer into Matrigel, these human lung organoids (HLO) form organised airway-like structures with both distal and proximal markers and supporting mesenchymal cells. A similar method to create HLO is reported by Leibel et al., [67] by directing key developmental pathways in induced human pluripotent stem cells (iPSCs). Additionally, Ramamoorthy et al., [68] have established a scaffold-free organoid system they term a “primitive lung-in-a-dish” (PLiD). The PLiD develops similarly to the less advanced single-cell spheroid models, but contains normal lung epithelial, endothelial, lymphatic, and fibroblast cells. The cells self-assemble into air-sac like structures and form organoids that are functionally similar to the human lung microenvironment. By introducing cancer cells to create a ‘metastatic tumour-in-a-dish’ (mTiD) they can also model interactions with cancer cells capable of undergoing lung metastases, such as breast cancer cells. Crucially, they demonstrate that the mTiD system can be used to study cancer cell colonization and assess changes to the metastatic microenvironment induced by cancer cells [68].

Despite advancements, organoid models still have some key limitations. For example, models such as the iPSC-derived organoid described above tend to be most representative of the human foetal lung as opposed to the adult lung making them less relevant for disease modelling. This is owing to the presence of alveolar progenitor cells; however, it is thought that this could be overcome by using primary lung epithelial cells. Additionally, like many other in vitro models, organoids lack the ability to recapitulate the recruitable immune system and other components of the native lungs such as innervation.

### Ex Vivo: Existing and Emerging Lung Explant Models

Ex vivo lung explant models aim to overcome the common limitations of in vitro assays. They mainly benefit from the ability to preserve the complex architecture of the lung tissue and as they are derived from whole tissue and can contain all the native cell types present within the lungs, including resident immune cells. Lung tissue explants can be generated from healthy, diseased, and even pre-malignant human and murine lung tissue and have been mainly utilised to study lung biology and respiratory diseases such as asthma [69], idiopathic pulmonary fibrosis [70], chronic obstructive pulmonary disorder [71] and infections [72] in pulmonary research to date. Whilst not yet widely adopted in cancer research, explant models may be relevant models not only to investigate primary lung cancers, but also cancers that metastasise to the lungs such as breast cancer.

The pulmonary metastasis assay (PuMA) is an ex vivo model that allows real-time assessment of metastatic progression in lung tissue explants. In this model, first established by Mendoza et al., [73] and described in Lizardo and Sorensen [74], fluorescent cancer cells are delivered via the tail-vein (TV) to allow lung seeding. After lung perfusion, the insufflated lungs are manually processed generating transverse slices which are maintained in ex vivo culture on haemostatic supports [74]. These lung slices are viable, with intact lung architecture. Using this model, Khan et al., [75] have demonstrated that genetic knockdown of lung microenvironment derived E-, L- and P-selectins results in significantly impaired metastatic progression of TNBC. The PuMA has also been used to identify and test novel therapeutic targets in other cancer models that undergo lung metastasis including Ewing sarcoma [76, 77], osteosarcoma [78] and melanoma [79].

Precision cut lung slices (PCLS) were first established by Dandurand et al., (1993) for pharmacological studies over 20 years ago [80]. The method is similar to the PuMA, however the lungs are precisely cut generating uniform slices between 100 and 500 µm thick [81]. As multiple slices can be generated from a single animal, PCLS reduce the number of animals needed to test hypotheses versus a traditional in vivo experiment [81]. In a recent proof of principle study focusing on chemoprevention in lung cancer, Sompel et al., [82] demonstrated that murine PCLS can recapitulate chemoprevention effects seen previously in vivo. Not only were PCLS able to accurately recapitulate the signalling pathways observed in vivo, but they were also used to identify prominent, resident immune cells within the lung microenvironment, and changes upon drug treatment. As with other in vitro and ex vivo model systems, PCLS are limited by the inability to study the recruitable immune system. However, the existing uses of PCLS in respiratory diseases and the ease with which the slices can be manipulated calls to question how the system could be used in cancer research. A possible approach could be to add exogenous compounds or cancer cells to PCLS to study the interactions between the cells and the lung microenvironment including their ability to invade, grow and change the tissue.

In other studies, Xiong et al., [83] created a decellularized murine lung matrix with preserved ECM composition to study the colonization of breast cancer cells. Demonstrating that metastatic breast cancer cell lines can adhere, grow, and colonize when introduced to the decellularized lung matrix, consistent with the in vivo phenotypes of the cell lines [83]. Methodologies also exist to perform whole lung perfusions [84] however the availability of human tissue is a major limiting factor to the uptake of this technique. For murine studies, optical tissue clearing now permits the imaging of intact, whole lungs via light-sheet microscopy. This has been previously used to study TNBC metastasis and chemosensitivity in murine lung tissue [85]. Ex vivo models offer notable advantages over many in vitro and in vivo systems including the ability to recapitulate the structural and cellular complexity of the tissue, and to reduce the number of animals required for a single experiment. However, they also have some important limitations yet to be addressed, such as the inability to study the recruitable immune system, which is where in vivo systems are necessary.

### In Vivo Models

In vivo models performed in mice are still considered to be the ‘gold standard’ in cancer research. These models benefit from greater physiological relevance than in vitro and ex vivo model systems as metastases develop within a living system. Orthotopic implantation (OI) and tail vein injection (TV) are the two most commonly used methods to study BCLuM in vivo [86]. The OI model involves the implantation of cell lines or patient derived xenografts into anatomically relevant sites in the animal. For the study of BCLuM, this involves injection into the mammary gland. In this model, lung metastatic lesions develop much later after establishment of a primary tumour. This contrasts with the TV model whereby injection of cancer cells via the TV results in immediate implantation in the lungs. A study conducted by Rashid et al., [86] used a genome wide microarray to demonstrate that there are no significant differences in gene expression between lung metastases of the 4T1 mammary carcinoma cell line produced via OI or TV [86]. Despite this, debate still exists around which model is the most appropriate to study BCLuM. The relative advantages and disadvantages of OI vs TV injection have been discussed extensively and are summarised in [86].

In advancement of these traditional in vivo techniques, and to further resolve the cellular interactions and alterations within metastatic niches, Ombrato et al., [87] engineered a labelling 4T1 cell line that following implantation in the lungs via TV injection, releases a cell-penetrating SLP-mCherry fluorescent protein to surrounding host cells within the tissue microenvironment. In the lung, they uncovered a unique population of stem-like cancer-associated parenchymal cells that contribute to metastatic niche formation [88]. A further development could involve the use of synthetic, scaffold implants. A recent study by Wang et al., [89] demonstrated that synthetic polycaprolactone scaffolds are well tolerated in mice, and create a metastatic niche similar to the endogenous murine lung. Interestingly, they demonstrate important differences in the phenotypes of immune cells that are recruited to the scaffold versus the lungs following the OI and colonization of 4T1 cells. Their synthetic niche appears to have dormant, anti-tumour activity compared to the lungs, implying a potential therapeutic benefit [89].

In vivo models hold a distinct advantage over the in vitro and ex vivo models discussed here owing to the ability to study tissue microenvironments and their interactions with cancer cells in a living system. However, whether the model incorporates native or synthetic microenvironments, there are some common and important limitations of all in vivo model systems. In vivo experiments are time-consuming, expensive to maintain and often require a lot of animals to interrogate a single hypothesis. Additionally, there is ongoing debate as to whether in vivo models accurately reflect clinical metastases, implying the models may be poor predictors of human disease progression.

Understanding the complexities of tissue microenvironments and metastasis, particularly in the context of BCLuM presents a significant challenge. Existing models used to study metastasis have both their respective strengths and limitations as discussed here. Learning from existing models in various fields of research, including respiratory diseases and other cancers that metastasise to the lungs, can inform the development of more accurate and representative models for studying BCLuM. Combining the strengths of different model systems and addressing their limitations will be crucial for advancing our knowledge of BCLuM and improving our ability to predict human disease progression.

## Liver Metastasis

The liver is the most common site of cancer metastasis and accounts for a quarter of all cases [90] largely due to the organ’s unique susceptibility to metastatic colonisation resulting from its dual blood supply [91]. Breast cancer liver metastasis (BCLiM) occurs in 50–70% of patients diagnosed with secondary breast cancer [92]. HER2-positive breast cancer preferentially colonises the liver [93] and, these aggressive metastases, result in a poor prognosis with median survival at 2–3 years. If untreated, with chemotherapy, endocrine targeted therapy or radiation, survival is limited to 4–8 months [94]. Hepatic failure, ascites, thrombosis in the portal vein and poor nutrition are the main complications leading to death [95]. Models of the liver microenvironment and the colonisation by breast cancer are limited.

### In Vitro Models: 2D, On-a-chip and Organoid Approaches

Precision cut liver slice culture has been developed to allow maintenance of the tumour micro-environment in an ex vivo culture [96]. Tumour samples from liver resection are cut using a Vibratome to produce 6 mm × 250 µm slices and cultured for 7 days. This technique has been shown to work well for numerous solid tumours, both primary and metastatic, but varying success and longevity of culture is an issue. A major drawback to this method is that slicing of liver tissue results in liver damage and cell death. Hepatocyte death results in differentiation of hepatic stellate cells into myofibroblast like cells which produce ECM and fibrosis [97].

3D modelling of the liver, both normal and cancerous, within an organoid culture relies on self-organising structures generated from stem cells, LGR5 + cells of iPSCs, that model the in vivo tissue from which it came. Patient derived organoids (PDO) offer the opportunity to study human cancer in vitro. Using needle biopsies or resected tumours from surgery, organoids can be successfully established in approximately 30% of cases [98].

Microfluidic cultures, consisting of channels which allow continual perfusion, offer the opportunity to design complex 3D environments to study the effects of flow, cell localisation and the influence of chemical gradients for example [99]. A number of these systems have been used to investigate steps in breast to liver metastasis. Liver-on-a-chip technology has allowed modelling of the liver premetastatic niche [100]. Study of the interactions between different cell types within the liver as well as with breast cancer before colonisation has shown that breast cancer derived extracellular vesicles can induce endothelial to mesenchymal transition, resulting in collapse of vessels to allow easy access to the tissue.

### In Vivo

Numerous papers report spontaneous liver metastasis in mice following xenograft growth within the mammary fat pad (MFP), but these models typically result in metastases at other sites too, often at a higher rate than in the liver. MDA-MB-435 cells, for example, have been shown to produce metastases in the lung in 55% of the models, LN in 15% and liver in only 10% [101] whilst the 4T1 mouse mammary tumour line spread to bone and visceral tissues in all cases [102]. Models involving human xenograft growth within immune compromised mice [103] offer the opportunity to investigate human derived BCLiM but due to immune suppression in the mice investigation of the role of the immune system is not possible. Models of BCLiM using syngeneic cell lines, such as 4T1, implanted into the orthotopic site allows assessment of metastasis within an immune competent environment but liver metastasis in these models is rare [104] and normally occurs with secondary growth at other sites [105].

Several methods have been developed to overcome these issues. Tumour explants from genetically engineered mice: tumours which have developed in K14CreECadf/fP53f/f mice, which model invasive lobular breast carcinoma, can be reimplanted in naïve hosts which develop orthotopic tumours. Following primary tumour resection, 18% of these mice develop liver metastasis [106]. Liver metastasis occurring spontaneously in genetically engineered mice is rare although some examples do exist including, for example, the H19-IGF2 model [107]

In another attempt to develop a model of liver metastasis, Rikhi et al., [108] performed direct injection of liver cells into the frontal liver lobe of a FRG™ KO [ Fah(-/-) Rag2(-/-)Il2r g (-/-)]) NOD mouse which has been shown previously to allow successful colonisation of human hepatocytes [109]. Co-injection with human hepatocytes had no influence of tumour growth so in this model MDA-MB-231 were injected alone. Tumours representative of human BCLiM were seen with vascular invasion and metastasis to the lungs and peritoneum.

To allow investigation of liver metastasis in a mouse with a full immune system and without multi-organ metastatic growth cells can be injected directly into the portal vein. These techniques were originally designed for colorectal [110] and melanoma [111] cell lines and they are now used for BCLiM [112]. Metastasis rates vary but highly metastatic 4T1 cells form within the first 40 days.

## Brain Metastasis

Patients diagnosed with breast cancer to brain metastasis (BCBrM) often face a particularly low life expectancy [113], distressing neurological symptoms, and a distinct lack of therapeutic options [114]. The brain is largely separated from the rest of the body by the blood–brain-barrier (BBB) and contains unique physical, cellular, and metabolic properties. This makes it an extremely unique environment for metastatic dissemination [115]. Historically, not a lot is known about this microenvironment in the context of metastatic disease. In recent years, however, the rapid development of new in vivo*, *ex vivo and in vitro models is now quickly changing this situation [116, 117]. This section aims to summarise the development and potential usage of these models as well as being transparent about the challenge’s researchers may face when using them.

### In Vitro Models: Microfluidic, On-a-chip and Organoid Approaches

In vitro models have the distinct advantage of being able to closely follow the interaction between cancer cells and their microenvironment. Not having to establish these models in an animal allows for less phenotypic variability, cheaper costs and less animal suffering [118]. The increasingly sophisticated models of these processes are now often an adequate replacement, and, in certain contexts, are preferred over in vivo models.

With dimensions exceeding no more than a few centimetres, microfluidic devices are now allowing researchers to create some of the most accurate models of the BBB currently seen [119]. The microfluidic BBB (μBBB) allows researchers to induce a flow state for cancer cells, providing shear stress and a 3D structure, significantly enhancing physiological relevance [120]. They usually provide real-time readouts and are more cost-effective than many other experimental models [121].

As summarised by Augustine et al., [122], microfluidic devices, an effective µBBB model, will generally contain the following components:

1. A vascular chamber, where the cells are initially present. The cells need to be in a flow state at the start of the experiment.
2. A selectively permeable barrier. This barrier is often produced from certain plastics such as polyethylene terephthalate, polyester or polytetrafluoroethylene with pore sizes ranging from 0.2 or 0.4μm in diameter. Hydrogels such as collagen are also used to simulate the ECM on these models.
3. Cellular components – ideally both a brain endothelial component, as well as other brain neurovascular cells such as astrocytes [116].
4. Biosensors (either optical or electrophysiological) for monitoring separate components of the BBB microenvironment [123].

Although µBBB models which model metastatic invasion into the brain are limited in number [124], the current models in circulation are very strong [122]. A well-known model has been produced by Xu et al., [125] who was able to produce a µBBB model using rat-derived astrocytes and brain microvascular endothelial cells (BMECs) – cultured on rat-tail type-1 collagen. Although not specific to breast cancer, Liu et al., [126] were able to produce a µBBB investigating the invasion of breast cancer cells to the brain. They utilised human astrocytes and human brain microvascular endothelial cells to recreate the BBB. For their noncellular components, they used PDMS as their plastic with a mixture of collagen I and fibronectin to mimic the ECM. Microfluidic BBB models have been used to investigate drug treatment in the context of BCBrM [122].

While these models are increasingly popular due to being physiologically accurate, they are highly specialised and difficult to set up. 3D printing technology is likely to help mitigate the problem but these are still challenging models. Finally, there is a lack of standardisation of these models most notably the quantification of their performance parameters [122].

The use of organoids allows research groups to investigate the behaviour of breast cancer cells in the context of the brain microenvironment in an in vitro setting. Organoid models have been used extensively when investigating glioblastoma [127], however, far fewer research groups have developed organoids for brain cancers arising from extracranial tumours.

Margarido et al. [128] established an organoid model by inducing primary ductal carcinomas in E-cadherin-mCFP reporter mice using the mouse mammary tumour virus—polyoma middle-T antigen (PyMT). The primary tumour, generated in the MFP, was transplanted into the brain. After six successive transplantations, organoids were derived from the resulting brain tumours. The organoids were then injected into the brain of other ROSA26mTmG FVB mice. This model has subsequently been used by Castro et al., [129] among other research groups indicating its strength and reliability.

Another method was utilised by Choe and Lee [130] who implanted MDA-MB-231(eGFP) cells into a 70-day old cerebral organoid developed from human endothelial stem cells. The model displayed evidence of interactions between the cancer cells and the cells developed from the cerebral organoid. Furthermore, there was an increase in proliferation of the cancer cells as well as an increase in markers for stem cell activity and epithelial to mesenchymal transition.

Patient-derived organoids from surgically resected BCBrM, have been cultured by Donzelli et al. [131]. They developed a brand-new method for cultivating organoids from patient metastatic material, which increased the success of their experiments. This method replicated the conditions of the ‘destination material’ of the metastatic tissue, instead of the ‘originating material’, which may provide a brand new basis for further experiments [131].

Organoids have been a large step forward in modelling BCBrM, but their lack of a vascular component as well immune cells or an ECM does severely limit their utility as models of metastasis. Finally, unlike microfluidic methods, it is often difficult to get ‘real-time’ data from the models [132]. Organoids however, are very strong models when testing the effects of genetic manipulation or drug models [132].

The transwell model is a powerful tool for mimicking the BBB and is a modification of the boyden chamber model. This involves seeding one or more brain-specific cells onto the semipermeable membrane to replicate the BBB with modifications to the bottom-portion of the chamber to mimic the brain microenvironment. A further modification involves the use of hydrogels to mimic the ECM within the brain.

There are multiple options for creating the endothelial component of the transwell model. Initially, research groups would use primary cells derived from patients, however, this process is slow, labour intensive and relies on researchers having access to clinical materials [133]. These cells would also rapidly lose brain-specific properties when culturing for even short periods of time, as well as there being a large amount of variability in the cellular properties from patient to patient [134]. Mouse or primate-derived cerebral endothelial cells have been used but many of the same issues still apply. What has recently occurred, however, is the development of brain microvascular endothelial cells (BMECs) from iPSCs [135]. This is typically done with the co-culture of these cells with pericytes [136]. Newly immortalised BMECs have also been used in other studies replicating the BBB [137], however, this has not yet been done for BBB specifically looking at BCBrM. This monocellular layer of cells can either be kept as a model in and of itself, or it can be further modified, where other brain-specific cells are added in co-culture [138].

Several groups have modified this model by placing brain-specific cells to the bottom layer of the insert such as astrocytes and pericytes [139]. Further modifications have included placing neuronal cells on the bottom of the cell-culture well itself in order to represent the brain microenvironment that the cells are migrating to. This method has been coined the ‘four-cell method’ by Stone et al., [140].

Using co-culture alongside a transwell insert has proven to be a step forward in modelling the BBB. The basement membrane, however, a key component of the blood–brain barrier is missing within these models. Culturing the brain-specific cell lines within gel-based scaffolds [141] reduces the time for cells to seed onto the transwell inserts, allowing for greater communication between the cells themselves, and provides a physical barrier, which reduces the permeability of the transwell insert. Matrigel is commonly used for this purpose [142]. However, Li et al., [143] suggest using a mixture of collagen I and IV, in order to enhance the integrity of the BBB models. A further development on the materials front has involved the use of hydrogels such as gelatin-methacryloyl, which further increases the physiological relevance of the BBB model.

While transwell models are becoming increasingly more complex and better able to represent the human BBB, major challenges still exist. Many of these models are unable to replicate the neuro-inflammatory environment that is often seen in cancer cell invasion to the brain. One limitation is that these models cannot be cultured for more than a week precluding long-term studies. Another is that the cells within the transwell model are never exposed to a flow-state and experience no sheer stress.

### Ex Vivo Techniques

The brain slice model is a method of growing cancer cells inside slices of a brain taken from a mouse in an ex vivo setting. This combines in vivo physiology with the advantages of in vitro work. The cancer cells are grown in and exposed to all of the primary cells of the mouse brain as opposed to only a few cell lines in other models, which greatly increases the physiological accuracy of the model. Furthermore, the fact that the work is performed ex vivo means that cell–cell interactions can be imaged via time-lapse microscopy [144]. With this method, many slices can be cultured from one brain. For example, Ciraku et al., [144] were able to culture 35–40 slices with each brain. This reduces the number of animals needed, making the experiments both cheaper and more ethical. These models also tend to be faster to produce than organoid models, which can be more useful for experiments that are time-limited or require high throughput.

Ciraku et al. [144] and Ferraro et al. [139] used nude mice to produce slices which bore MB-MDA-231, BT474 cells or MDA-MB-361 cells. Both groups established these models using intracranial injection. Zhu et al. [145] established a syngeneic mouse model using intracardiac injection of E0771-BrM cells.

Slice models have also been established without needing to implant the cell lines directly into the mice. Using the brains of neonatal mice, it is possible to establish a co-culture system between brain slices and MCF-7 cell lines [146]. Louie et al., [147] performed ex vivo implantation by seeding both MDA-MB-231 and MDA-MB-361 on top of the brain slices developed from p8-10 C57BL mice.

There are limits associated with slice cultures. Firstly, the cells do not need to cross any endothelial barriers to invade and colonise the brain. Therefore, these models can be great for measuring proliferation but may be inappropriate as a replica of the metastatic cascade. Secondly, the models mentioned only use breast cancer cell lines, which means that although the microenvironment is very genetically diverse, the tumours themselves are very homogenous, reducing the physiological relevance of the model. Finally, there is a limited period of around 7–14 days for which the slices can be kept in culture, meaning that these experiments are usually only appropriate for short term studies.

### In Vivo

Despite the recent developments of in vitro and ex vivo models, in vivo work remains the ‘gold-standard’ when investigating BCBrM. A number of animal models are now available and selecting the correct model is highly dependent on the context of the experiment being carried out [117]. This review will mainly focus on mouse models for BCBrM, but the use of other animals such as rats, drosophila or zebrafish is becoming increasingly commonplace [148, 149].

There are generally three categories of implantation site available to researchers: orthotopic, intraventricular/intracarotid and intracranial [150]. Selecting the best implantation site is highly dependent on the context of the experiment being conducted.

Orthotopic injection in the MFP to produce BCBrM has been successfully carried out using GFP–4T1-BrM5 cells in BALB/C mice [151] and MDA-MB-231 (mCherry) cells in NSG mice [152]. The initial technique is relatively simple, and the mice are rarely harmed by procedure. This is the most physiologically accurate model as the tumour cells go through all stages of the metastatic cascade [153]. Cancer cells injected into the MFP, however, do not always metastasise to the brain and will almost always metastasise to extracranial sites such as the lungs [154]. This often risks the mice having to be sacrificed early during experiments. It is extremely difficult to produce tumours of similar sizes using this technique and this method often takes a lot longer than other implantation techniques. This is a particularly useful technique to investigate the impact that certain gene aberrations or therapeutics at early stages of BCBrM [153].

Implanting cancer cells into the left cardiac ventricle is commonly used to produce BCBrM [155, 156]. These models often have a much shorter metastatic latency than orthotopically injected mice [156]. Brain metastases are also more common than with orthotopic injection, particularly when brain-tropic cell lines are used, and produce a more reproducible median survival. Issues with this method include variable rates of cranial metastatic spread, the formation of extracranial metastases, and the cancer naïve host which differs from patients with BCBrM [157]. This issue could be addressed by systemic inoculation alongside orthotopic injection.

Injecting cancer cells directly into the artery that supplies the brain dramatically reduces the number of extracranial metastases that form and increases the chance of tumours forming within the brain, whilst letting the cells go through the metastatic cascade. This method, however, faces similar issues to intracardiac inoculation such as introducing cancer into a naïve host and not modelling intravasation. Finally, this is a very difficult surgery, requiring a lot of specialised equipment and involves a lot of risk to the mice themselves [158].

Intracranial injection involves directly injecting cancer cells into the brain. Using this technique is a great way to produce large tumours with more control over their size at known locations in the brain This method requires a large amount of training to carry out and a lot of specialist equipment. Usually, only one, large tumour is produced which is not representative of what occurs in the clinic. The technique itself is associated with a high mortality, and even under the best conditions, mice do not survive long after successful implantation, potentially making it less useful for studying long-term treatment. The technique is the least physiologically relevant model; Inserting a needle into a mouse brain also produces an extremely inflammatory environment and introduces a severe disruption to the BBB [159]. This technique is often useful for high-throughput experiments, which are only designed to measure the growth of cancer cells in the brain and not invasion.

Syngeneic models, where mouse cell lines are used, allow researchers to use immunocompetent mice in their work which markedly improves the physiological relevance to any study being conducted [160]. This is particularly true if the model is used to investigate the microenvironment of the mice. These animals, are cheaper and easier to care for than their immunodeficient counterparts [161].

However, there are limitations associated with these models, particularly when murine cell lines are used. There are generally far fewer murine cell lines that are available than human, especially those which preferentially metastasise to the brain [117]. Daphu et al., [162] found that the genes associated with BCBrMs discovered in murine cancer cells used in mouse models, had very little overlap to the genes associated with BCBrM in the clinic. The immune system of mice has key differences compared to humans [163], which means that discoveries made regarding the immune system of the mice, in the context of cancer, may not be entirely applicable to humans.

Immunodeficient mouse models, such as NSG, athymic nude, or NOD/SCID mice, are commonly used in studying BCBrM [164]. Their lack of a functional immune system allows for the creation of xenograft models using either cancer cell lines or patient-derived cancers, forming more physiologically accurate tumours [165]. The use of human-derived cancer cells, either cell lines or primary cells will allow researchers to study the effect of human-specific drugs on the cells. The inclusion of PDX models, means that the tumours still retain their original microenvironment, for example, the stromal cells and ECM. Furthermore, there is a greater certainty that cancer cells will develop and the speed of tumour development is generally greater [166].

## Concluding Remarks

**Fig. 2:**
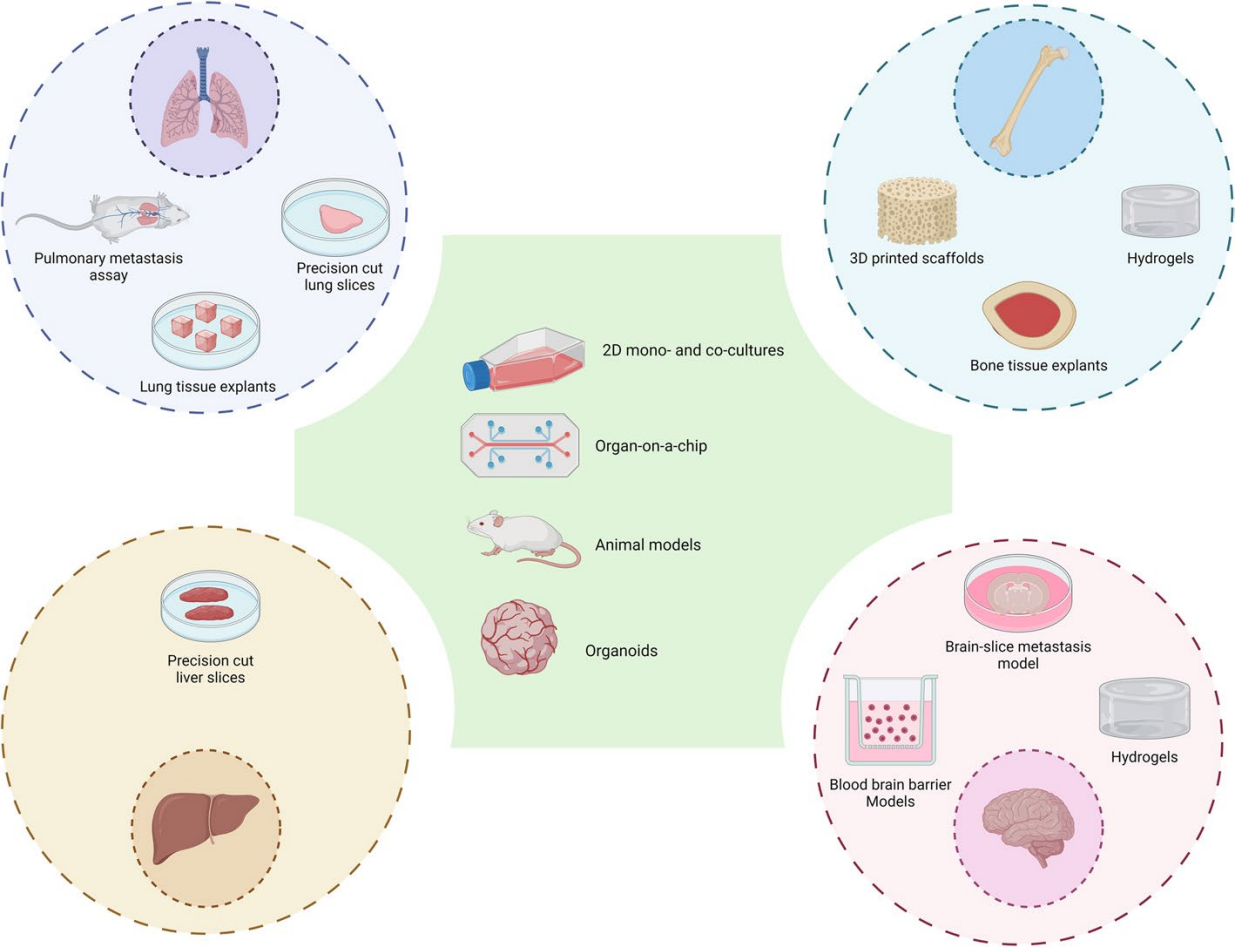
Overview of Different in Vivo, in Vitro, and Ex Vivo Approaches for the Study of Metastatic Microenvironments in Breast Cancer

There are numerous shared and distinct in vitro, ex vivo and in vivo (mostly in mouse) approaches that encompass different tissue components of bone, lung, liver, and brain metastatic sites (Fig. 2). These approaches attempt to model the interactions between the metastatic tumour and the tissue cells and investigate the effects of reciprocal communication between them on the cell biology, signalling pathways and ultimately drug responses. There are clear gaps in knowledge such as evolution of tumour cells in different metastatic sites, even in the same patient. Other gaps include models of rarer sites of metastasis such as the leptomeninges, different areas of bone other than the marrow and those common in lobular breast cancers such as the ovaries, gastrointestinal tract and peritoneum. In addition, there are rarer forms of breast cancer such as those in men and histological sub-types for which there is little literature about common types of metastasis. It will be essential that better models of the common sites of metastasis and those less frequent be further developed in the future to provide platforms for further elucidation of the ‘seed and soil’ concept and for the rational testing of therapies for advanced metastatic breast cancers.

## Data Availability

No datasets were generated or analysed during the current study.
